# Negotiating Novelty: Constructing the Novel within Scientific Accounts of Epigenetics

**DOI:** 10.1177/0038038520954752

**Published:** 2020-11-17

**Authors:** Martyn Pickersgill

**Affiliations:** The University of Edinburgh, UK

**Keywords:** biomedicine, epigenetics, novelty

## Abstract

Epigenetics is regarded by many as a compelling domain of biomedicine. The purported novelty of epigenetics has begun to have various societal ramifications, particularly in relation to processes of responsibilisation. Within sociology, it has stimulated hopeful debate about conceptual rapprochements between the biomedical and social sciences. This article is concerned with *how* novelty is socially produced and negotiated. The article engages directly with scientists’ talk and writings about epigenetics (as process and field of study). I aim to advance an explicitly sociological analysis about the novelty of epigenetics that underscores its social production rather than an account which participates in its reification. I attend to definitional skirmishes, comparisons with genetics, excitement and intrigue, and considerations of the ethical dimensions of epigenetics. Any assertions that epigenetics is exciting or important should not inadvertently elide reflexive consideration of how such characterisations might be part of the machinery by which they become real.

## Introduction

Epigenetic research is increasingly presented as a compelling domain of biomedicine, even if characterising it is not straightforward. Commonly, though, it is regarded as a field of study concerned with heritable changes to gene expression that do not change the DNA sequence itself. Epigenetics is also an emotional accelerant, with excitement about its implications for biology and medicine readily apparent within biomedical literatures (e.g. Allis and Jenuwein, 2016: 487; Rodríguez-Paredes and Esteller, 2011: 330). Enthusiasm is also evident in sociological scholarship; in this journal, for instance, Meloni (2014: 732) has argued that epigenetics can help to constitute ‘a more pluralistic and contingent vision of “the biological”’ (2014: 742). Rather than the banal ‘importing of biological knowledge into the social’ (2014: 742), such a vision is judged as having the potential to facilitate more fruitful interactions between biologists and sociologists. Elsewhere, the centrality of ‘the environment’ to epigenetics has been implied to underscore the import of sociology and the expertise of sociologists. For example, Rose (2013: 18) has described how ‘at its best, the turn to epigenetics marks a recognition of the inseparability of vitality and milieu which could give a crucial role for the social and human sciences in accounting for the shaping of vitality at the molecular level’. Sociological hopes for epigenetics, then, are often high.

This article engages with scientists’ talk and writings about epigenetics (as process and field). It aims to advance a sociological analysis about the novelty of epigenetics that underscores its social production. In what follows, I describe the backdrop to my arguments, outline the study from which my data emerged, and document some of the different ways through which the novel is synthesised and negotiated within scientific discourse. These include through definitional debate, comparisons with genetics, and considerations of the ethical dimensions of epigenetics. I seek to add to sociological deliberations about ostensibly innovative entities and practices through underscoring that newness should not be considered only as a pivot for analysis, but that this is also an important problematic in itself. I thus elucidate epigenetics as a test-case in the sociology of novelty.

## Social Science, Epigenetics and the Negotiation of Novelty

Interest from humanities and social science scholars in theorising about and with epigenetic knowledge and processes has been growing in recent years (e.g. Meloni, 2014; Papadopoulos, 2011; Rose, 2013). Some take the development of research in epigenetics to represent ‘a new opportunity for dialogue’ between social and life scientists (Meloni, 2014: 731). The potential for this relates to how ‘the environment’ has been perceived to be of enhanced significance within biomedicine, as a diversely factorialised progenitor for epigenetic changes that can have phenotypic effects (Landecker, 2011). This has proved compelling to both biomedical and social scientists who are concerned with how substances and processes beyond the boundary of the body might exert bodily effects through epigenetic action (Meloni and Testa, 2014: 431). For Nicolosi and Ruivenkamp (2012: 318), epigenetics ‘could be a new paradigm’ with the potential to ‘support humanities [scholars] and social scientists in their analyses and interventions’. In sum, a range of analysts have considered epigenetics as a potentially novel and important field that could ‘revitalise’ (Fitzgerald et al., 2016) sociology and social theory.

Other work has focused more on how epigenetics circulates within (Robison, 2016; Stelmach and Nerlich, 2015; Waggoner and Uller, 2015) – and, to an extent, impacts (Niewöhner et al., 2011; Pickersgill, 2018; Warin et al., 2020) – societies. Here, reproductive science and maternal health have formed a significant focus. In particular, analysts have shown how epigenetics can (or could) be leveraged to create enhanced responsibilities on women during pregnancy (e.g. Pentecost, 2018; Richardson, 2015; Valdez, 2018). Biomedical research on rodents is a key surface of emergence for a logic of epigenetic responsibility. Within this, ‘commonsense assumptions’ regarding sex and gender are constitutive of research that ‘illustrate[s] rather than interrogate[s] existing stereotypes about maternal agency and responsibility’ (Kenney and Müller, 2017: 23). As epigenetic knowledge moves beyond the laboratory, it can participate in ‘producing new versions of vulnerable, plastic life that require protection now’ – including prior to conception (Mansfield, 2017: 355). Although ‘the pre-pregnancy care literature predated the emergence of epigenetics as a popular scientific topic’ (Waggoner, 2017: 20), epigenetic ideas seem increasingly folded into constructions of women as responsible for foetuses not yet even conceived. When epigenetics collides with maternal health, the purported novelty of epigenetics often supports processes of responsibilisation and individualisation.

Sociologists and anthropologists have also considered the social dimensions of epigenetic research itself. For example, analyses of scientific discourse, interviews with scientists and ethnographies of laboratories have cast new light on the material-semiotic practices constituting epigenetics (e.g. Lappé, 2018; Lloyd and Raikhel, 2018; Niewöhner, 2015). Different areas of epigenetic research can inspire distinct biopolitical visions of the future; hence, the implications projected by scientists about their work are not epiphenomena of epigenetics writ large but relate to how epigenetics is enfolded within particular biomedical traditions (Müller and Samaras, 2018). For instance, Rapp’s (2011) ethnographic work on scientists studying the ‘epigenetic effects of social stress and paternal age on children’s mental disorders’ (2011: 668) shows how the idiom of epigenetics fosters a rich(er) imaginary of ‘the environment’, enjoining the ‘appreciation of complexity and nondeterminism’ in psychiatric aetiology (2011: 669). It is precisely this broadening of biologists’ conceptions of ‘the environment’ and ‘the social’ that have proven so compelling to some social scientists (Niewöhner and Lock, 2018). Elsewhere, though, analysts have been more concerned about how epigenetics can co-exist with and might augment longstanding forms of biological reductionism and determinism (Saldaña-Tejeda and Wade, 2019).

## The Sociology of Novelty

Be they optimistic or critical, commentaries on epigenetics – including some from social scientists – are commonly premised on a perception that the field represents something particularly new and important, stimulating excitement or concern. Yet, biologists themselves can sometimes be reticent to ascribe novelty to epigenetics (e.g. Niewöhner, 2011). Tolwinski’s (2013) study is notable for its attention to how scientists judge the import of epigenetics. She details a spectrum of opinion, which ‘suggests a far more complex and contested trajectory’ for epigenetics research than is often considered in external appraisals (Tolwinski, 2013: 366; see relatedly Pickersgill, 2016). Accordingly, the epistemic novelty of epigenetics cannot be taken for granted; rather, it is discursively negotiated.

As Webster (2002, 2005) has indicated, the presentation of objects as ‘old’ or ‘new’ depends in part on what work different social actors seek to achieve through various kinds of positioning. In other words, novelty is not only in the eye of the beholder, but its characterisation is also dependent on where others have placed the entity or practice under appraisal. Hence, not everyone will everywhere and always judge it as novel. If the nature of ‘the novel’ is contestable, then a sociology of novelty is possible (Pickersgill, 2019). My approach takes as its starting point the premise that novelty is not a fixed property of an object, theory or form of social action: it is, instead, an achievement worthy of study. The analytic gambit advanced in this article is influenced by the sociology of scientific knowledge (Barnes, 1983). Consequently, I engage with more materialist histories and sociologies of science that focus on the emergence of technoscientific praxis (e.g. Rheinberger, 1997) while nevertheless having a somewhat different contribution in mind.

My contention in relation to novelty, though, is not a radical (or perhaps ‘novel’) proposition. Sociologists are largely comfortable with the notion that scientific endeavour (Longino, 1990) and the constitution of knowledge (Bloor, 1991 [1976]) are social accomplishments operative within particular epistemic cultures (Knorr-Cetina, 1999), familiar with the idea that framing technoscientific developments as compelling can contribute to sociotechnical change (Fujimura, 1988), and accepting of the proposition that ontologies are negotiated rather than pre-given (Law, 1999; Mol, 1999). It should not be controversial to regard characterisations of the processes and products of science as mutable rather than quintessential, including when those characterisations are of originality and import (cf. Arribas-Ayllon et al., 2010, on ‘complexity’). Work on the sociology of expectations about innovation comes close to this, through analysis of the promissory discourse that attributions of novelty can inspire (Borup et al., 2006; Hedgecoe and Martin, 2003). Still, sociological attention rarely fixes specifically upon discourses and negotiations of novelty. I argue that centring the novel within sociology can contribute to our understandings of the dynamics of contemporary technoscientific praxis, and potentially further enhance the reflexivity of sociological encounters with biomedicine.

## Methods

This article draws from a qualitative project entitled ‘Epigenetics, Ethics and Society: Accounting for Responsibility in the Biomedical Sciences’, funded by the Wellcome Trust. This involved three intersecting approaches. Specifically, I: conducted 10 semi-structured interviews with senior (personal or endowed chairs) UK-based scientists undertaking leading work on (or on issues closely related to) epigenetics; undertook informal conversations with biologists in the USA, UK and Canada (including postdoctoral researchers, mid-career scientists and long-established professors); and examined scientific texts around epigenetics (particularly editorials, commentaries and review articles).

My work was motivated by two concerns. First, I was intrigued as to how practitioners of an area of science that has been regarded by so many as exciting and important judged it themselves. Hence, my sociological interest in epigenetics was focused on the enthusiasm it enjoined, not the science itself. Second, I wanted to explore how scientists working in a field regarded as having ramifications for healthcare and beyond delineated the implications of their work, and their responsibilities to wider society.

This article developed primarily from the interviews. I specifically sought out scientists who might be considered opinion-leaders, drawing up a list of potential interviewees based on markers of prestige such as significant grants from major funders, presence on relevant editorial boards and highly cited articles. I was interested in how scientists who could reasonably be regarded as influential players in the field of epigenetics sought to present, describe and inform it. The interviews ranged across definitional aspects of epigenetics, its normative implications and public and policy interactions.

Following informed consent, interviews were recorded and later professionally transcribed.^1^ The interview data then re-orientated my targeted engagement with the scientific literature on epigenetics, and served as vignettes for further conversations with biomedical researchers – shaping my analysis of the interviews. This was itself abductive in orientation (Timmermans and Tavory, 2014), with data aggregated into broad themes (e.g. ‘therapies’, ‘public engagement’ and ‘policy interactions’), that facilitated its closer interrogation in dialogue with wider sociological (and anthropological) literatures.

While this article emerged as a consequence of the analysis as described, it is also informed both by my informal conversations with scientists and by my attention to the biomedical literature. In the former case, I spoke to a range of faculty (postdoctoral researchers, lecturers and professors) across several universities in three countries. These were a mix of serendipitous conversations by people finding their way into epigenetics research, chats over coffee with scientists arranged in part to discuss their research and my interests in epigenetics and society and specific meetings where the aim was explicitly to seek wider opinion from internationally regarded experts during which I made notes. In the latter case, I consulted opinion pieces and research papers written by my respondents, other key papers that garnered attention (including but not limited to citations), and editorials and review articles in key journals. I also undertook targeted literature searches to further explore themes that had emerged in the talk or texts of my interlocutors.

My analysis will inevitably have been inflected by my ongoing position as a sociologist with research management responsibilities in a large medical school. Many of my everyday professional activities constitute a form of fieldwork that is revealing of how knowledge production and adjudication occur within biomedical milieus, shaping how I read and respond to interview data with scientists. While it is challenging to narrate how, exactly, this ongoing interaction with biomedical scientists in professional and personal life has shaped my work, it seems unlikely that this is irrelevant to how I understand and treat scientific novelty.

## Characterising Epigenetics

As noted above, although epigenetics is commonly defined as heritable changes to gene expression which do not go on to change the DNA sequence itself, more precise meanings remain contested (Landecker and Panofsky, 2013; Meloni and Testa, 2014; Pickersgill, 2016). I want to dwell on this point: partly, because of its centrality to scientific discourse around epigenetics; also, though, because I suggest that strategies to bound epigenetics through the ongoing articulation of definitions perform ‘novelty work’. Specifically, they reflect and participate in propelling an understanding of the field as new and significant. In what follows, I am less concerned with the particularities of those definitions, so much as with the fact of their diversity.

Characterisations of epigenetics are located between axes of capaciousness and restriction. Some allow for a range of processes and fields to be categorised as epigenetic(s); for instance, emphasising processes and mechanisms that are not necessarily heritable, such as transient histone modification (Bird, 2007). Others, though, involve more careful boundary work (Gieryn, 1983) in order to advance stricter demarcations of the field that often privilege heritability (Deans and Maggert, 2015). My interviews reflected this debate, as indicated by two quotes below regarding the role of chromatin (i.e. a ‘package’ of DNA, RNA and protein) within epigenetics. R5, for example, drew comparisons between himself and other scientists, and advocated a certain ontological openness:

[P]eople get grumpy sometimes when you go to chromatin meetings and lots of people in the chromatin world now will start talking about epigenetics and their research will be badged as epigenetic. Well, I have no problem with that. I think that’s fine.

R6, however, took a harder line on who could lay claim to be working in epigenetics: ‘[S]ome people call themselves an epigeneticist because they work on modified DNA and modified chromatin. That’s nonsense. What they’re working on is modification. They’re not working in epigenetics.’ Within the bioscientific literature, researchers have problematised the definitional heterogeneity of epigenetics (see Bird, 2007; Heard and Martienssen, 2014; Holliday, 2006; Isles, 2015). Some have sought to domesticate diversity (e.g. Deans and Maggert, 2015; Greally, 2018), such as by amalgamating different characterisations and parsing important common features. Rather than constituting the unanimity many commentators urge, this has multiplied the definitions available. Each one in turn contributes to the notion that epigenetics is sufficiently new and important to necessitate such debate.

While the scientific ‘problems’ relating ‘to these definitional ambiguities’ (Deans and Maggert, 2015: 887) might be regularly lamented, a further corollary is that locating the implications of epigenetics for health and society can be challenging. This seems not to have limited bioethical research on epigenetics (Huang and King, 2018). Nevertheless, it is at least potentially an issue for scientists when asked to comment on the ethical dimensions of epigenetics: ‘[I]f you want to discuss about the implication of something you have first to define the boundary of this something. And today, this boundary is exploding, we have absolutely no idea what epigenetic[s] means today’ (R3). In practice, however, scientists continue their research, and flexibly characterise work as epigenetics (or not) in bids, presentations and articles (cf. Barnes and Dupré, 2008; Fox Keller, 2000; Rheinberger, 2010). Indeed, immediately after making the remarks above, R3 stated: ‘probably the best definition is something that modifies expression of genes, not based on the change in DNA’. Through their comments, R3 illustrated how feasible it is for scientists to speak across different registers when discussing epigenetics. Specifically, they deployed a kind of contingent repertoire to underscore the uncertainties around what epigenetics really is, while participating in a more empiricist repertoire that stabilised the nature of epigenetics for the purposes of undertaking and discussing research (cf. Gilbert and Mulkay, 1984). By shifting between registers, scientists are able to develop studies in epigenetics while also reflecting on terminological instability.

Epigenetics is thus, to an extent, in flux: both what this research field properly comprises, and what epigenetic mechanisms are, remain open to debate. That frequent attempts are made to close this down is a reminder of what is at stake for those who succeed in taming contestation to accord with their own position. One key dividend is the possibility of enhancing some research agendas over alternatives, and perhaps attracting capital to certain projects while restricting its availability for others (Calvert, 2006; Schyfter and Calvert, 2015).

Consequently, practices of promoting, defending and attacking different conceptions of epigenetics do not necessarily reflect the fact that it is an inherently important and novel field, the boundaries of which require demarcation and fortification for its nurturance. Rather, we can read the proliferation of definitions as one means by which this novelty itself comes to be produced and propelled. The processes of problematisation through which the ontology of epigenetics is reflexively unsettled and carefully rebuilt present the field as worthy of such epistemic (as well as economic and emotional) investment. Hence, debate around demarcation within epigenetics discourse contributes to presenting the field as holding a wider significance than biomedical traditions within which ‘definitional ambiguities’ (Deans and Maggert, 2015: 887) and skirmishes are less routinely staged or publicly visible (even while they continue to exist, as in genetics; Barnes and Dupré, 2008; Fox Keller, 2000; Rheinberger, 2010).

## Genetics and Epigenetics

The presentation of newly consolidating biomedical realms as pioneering and innovative is one means by which expectations are generated, scientific (and wider) intrigue fostered and capital attracted (Calvert and Fujimura, 2009; Hilgartner, 2015). In epigenetics, journal research articles, commentaries and editorials co-construct the novelty of the field through, for instance, accentuating how epigenetic processes add another layer of complexity to understandings of genetics. A common trope in this regard is to foreground the definitional salience of the Greek prefix *epi-*, where the root of this as (e.g.) ‘upon’ or ‘over’ is emphasised. An indicative editorial in the journal *Nature* simultaneously acknowledged the import of genetics while asserting the need for new studies into epigenetics:

It is hard to think of any branch of human biology that has not benefited from the human genome sequence [. . .] *But despite the progress, each question that the genome helps to answer throws up further questions*. Much remains to be understood about how genetic information is interpreted by the individual cells in our body. *This is where epigenetics comes in*. Upon the genome, on the genome, over the genome – take your pick – epigenetics collectively describes changes in the regulation of gene expression that can be passed on to a cell’s progeny but are not due to changes to the nucleotide sequence of the gene. (Anonymous, 2015: 273; paragraph breaks removed, emphases added)

By noting that epigenetics focuses on an additional layer of complexity, it can thus be framed as redressing the limitations of genetics: that is, epigenetics can answer questions that genetics cannot. This framing occurred in some of my interviews and wider conversations with scientists; for example, R4 phrased the shortcomings of genetic science in terms of the somatic uncertainties that persisted even after much attention to ‘the genetic components’ of diseases:

I hope, and I don’t know whether that’s going to pan out, that a lot of the common adult diseases in humans, you know, in which the genetic components are better defined now, but a lot of the causation is missing, so to speak, taking all the genetic factors into account, that the important epigenetic components could be identified and ultimately therefore converted into new treatment ideas.

A similar temporo-epistemic hierarchy (i.e. ‘first genetics, then epigenetics’) was presented by R3:

[T]he way the environment act[s] on gene is through epigenetics, so if you want to analyse, to *really* analyse the effect of the environment, you have to develop some epigenetic studies and to think about the consequence of what you will find. Because today, er it’s interesting because er genetics used to be Mendel and his peas. And then in the last 30 years, everything has become genetics. And I *think*, er and we see the limitation of that, and I think that in the next 20 years, everything will be epigenetics.^2^

This hierarchy was not universal, however. Some scientists I spoke to were hostile towards the epistemic ostentatiousness of a discourse that presented genetics as yesterday’s news (Pickersgill, 2016). Further, review articles (e.g. on cancer and epigenetics) often intertwine talk of genes and genetics with discussion of epigenetic processes. Such articles reinforce the mutual significance of genetics and epigenetics for the discovery of new drugs (e.g. Feinberg et al., 2016; Jones et al., 2016; Plass et al., 2013). In one, published in *Nature Reviews Genetics*, Plass and colleagues (2013: 765) argued that ‘high-resolution genome-sequencing efforts have discovered a wealth of mutations in genes encoding epigenetic regulators that have roles as “writers”, “readers” or “editors” of DNA methylation and/or chromatin states’. Such mutations ‘have the potential to deregulate hundreds of targeted genes genome wide’. Ultimately, a better understanding of these processes will ‘inform novel therapeutic strategies’. We can see, then, that genetic science and technologies are conceived of as essential for comprehending how epigenetic effects might be exerted in the first place, and to what ends. Notwithstanding this, the salient novelty in articles like those by Plass et al. lies in epigenetic processes and the possibilities that exist for innovations which might modify them.

To summarise, the novelty of epigenetics is, in part, constituted both through texts and talk explicitly concerned with epigenetic process which present genetic work as having limits that research in epigenetics can transcend (see relatedly Arribas-Ayllon et al., 2010). It is also generated through writings aimed at readers such as scientists working in oncology, wherein epigenetics is introduced as a means of indicating thrilling new possibilities and hence configuring the field of epigenetics as exciting and new in itself by virtue of that which it enables.

## Excitement and Innovation

Whatever epigenetics is, it is certainly ‘a fashionable subject’ (Deichmann, 2016: 249). All the scientists I spoke with were well aware of this modishness. Prior to agreeing to an interview, one quizzed me over email over what I thought epigenetics was. I assumed that this ‘test’ sought to assess the acceptability of my motivations for requesting an interview: was I merely jumping on the latest bandwagon?^3^ Some researchers I interviewed were positive about what they described as the expansion of epigenetics. R4, for instance, was ‘very excited about the science’. They went on to say:

I think we are still at the stage where it’s [epigenetics] expanding quite a lot. I think we’re now in a much better position in terms of understanding, truly understanding the mechanisms. I think we’re beginning to develop towards where more specific intervention is possible. That’s very exciting, I think for medicine. And we’re seeing this enormous spread of epigenetic knowledge into other areas of biomedicine

Other scientists (both my interlocutors and journal commentators) painted a more modest picture of the rise of epigenetics. Still, its enhanced prominence within biomedicine has been often discussed and/or advocated through, for instance, scientific review articles (e.g. Dawson and Kouzarides, 2012; Feinberg, 2018; Heyn and Esteller, 2012; Millan, 2013). This textual form is particularly important for field-construction and conveying scientific promise (Arribas-Ayllon et al., 2010; Hedgecoe, 2006; Myers, 1991). In our conversation, R7 described the rise of interest in epigenetics in their own area of specialism: ‘when you go to cancer or ageing meetings now, there’ll probably be an epigenetic session. Whereas 10 years ago that wouldn’t have been the case.’ As they put it: ‘almost without noticing it I guess epigenetics of cancer is now really, really *hot*’ (cf. Fujimura, 1988).

For one interviewee (R5) who spoke largely about histone modification, research in epigenetics was ‘so *exciting* for medics’ and ‘the general public’ because it emphasised the environment:

[Epigenetics] appeals to, I hope will start to appeal to the general public, although we’ve got some work to do there because it gives a mechanism for how your, as I say, your diet, your lifestyle, you know, all the rest of it can feed into your, into your genome and, you know, we’re beginning to understand how these things can have a very *profound* effect on the way your body works.^4^

Some of my respondents presented epigenetics as potentially holding implications for future health, and the catalysis of innovation. This promissory aspect was especially apparent vis-a-vis cancer, and in the preponderance of writings highlighting drug-discovery targets for therapies acting upon epigenetic mechanisms (e.g. Dawson and Kouzarides, 2012; Garraway and Lander, 2013; Jones et al., 2016). R3, for instance, suggested that epigenetic effects could be used as a biomarker for carcinogenic pollutants:

[C]hemicals can modify your epigenetic, the signature epigenetic of your genes. So, why I say that is because there’s some question about pollution, and er the effect[s] of a lot of pollutant[s] which we know have some effect[s], different kind of effect[s] on cancer and how they can also modulate, er the ways a gene function[s] through some epigenetic change. So I think that in this regard it’s quite, epigenetic[s] can be a way to analyse the effect of the change of the environment, and the danger of the environment.

R5 felt that innovation was already happening:

[T]he medics obviously have come in, you know, very, sort of, willingly and rapidly, because both the enzymes and the binding proteins offer an array [of] potential drug targets. And these are now being used and some are finding their way [into the] clinic. So it’s something that appeals to pharmaceutical companies, ’cause of drug development, it appeals to the medics, ’cause it *is* generating new therapies. I mean it’s *always* slow, but it’s starting to feed through.

Despite R5’s optimism, other respondents expressed various kinds of unease about the notion that epigenetic research held straightforward implications for healthcare and society. In relation to oncology, R6 argued that although cancer was an area of drug discovery regarding epigenetics, the direction of current therapeutic innovation pathways might be flawed. R8 reflected critically on what they saw as erroneous perspectives about biopsychosocial causality, and hence the limits of epigenetics:

[T]here’s a lot of stuff now about maternal diet and how that influences your foetus. You know it probably *does* influence your foetus because your foetus is growing in your body, if you’re a mother, but the idea that it’s writing long term information on your DNA, er I think is a little bit insidious. It sort of gives this erm kind of slightly sinister er message. So, *out there*, there’s quite a lot of interest in the idea of epigenetics, I think, you know, in psychiatry and things like that, people love the idea that there’s a kind of *molecular* mechanism underlying a lot of the things they see. But it’s not clear to me it’s true.

Accordingly, some biologists I spoke with were circumspect about the innovative potential of epigenetics, the claims made about the science, and the expectations with which it is associated. Even R4, who as noted earlier spoke of excitement about epigenetic research, also asserted: ‘we have to be very cautious as scientists to portray the science appropriately, both in terms of the excitement and the *promise*, but also in terms of the *limits* of knowledge’.

Excitement about and interest in epigenetics was thus something that my respondents either expressed directly or ascribed in others. While emotive responses like excitement are key features of scientific praxis (Fortun, 2015; White, 2009), they are not inevitable. In the case of epigenetics, the purported novelty of knowledge produced in the field may go some way to explaining the excitement associated with it. This is not least because university science is a profession that is in large part organised around a trope that the new should generally (if not always) inspire enthusiasm (Fujimura, 1988). At the same time, the data above demonstrate how the purported novelty of epigenetics can also propel unease and dissatisfaction by scientists who resist characterisations of epigenetics as representative of epistemic innovation.

## Ethics and Novelty

One discursive site for the generation of excitement and expectations around purportedly novel technoscience is the bioethics literature (Hedgecoe and Martin, 2003). Exhilaration and anticipation are clear within the first sentence of the abstract for one early commentary: ‘epigenetics is one of the most scientifically important, and legally and ethically significant, cutting-edge subjects of scientific discovery’ (Rothstein et al., 2009: 1). Foreshadowing a burgeoning bioethical literature (reviewed by Huang and King, 2018), Rothstein and colleagues framed epigenetics as significant for issues such as environmental justice and intergenerational equity. In this section, I consider how scientists themselves construct the ethical dimensions and implications of epigenetics.

Importantly, my respondents did not generally conclude that there were immediate ethical ramifications distinct to epigenetics. As R8 put it: ‘In a way *genetics* raises more ethical problems than epigenetics *ever* could.’ When asked about the ethics of epigenetics as compared to genetics, R5 noted: ‘I can’t *think* of anything offhand that you could badge as epigenetics that is going to, that is, would generate *concern*.’ Hence, we can see that the (ethical) novelty of epigenetics is not only assembled through talk and texts – it can be deconstructed as well. This underscores the negotiability of ‘the novel’.

When prompted, my interviewees eventually advanced some form of reflection on the implications of epigenetics. However, I sometimes experienced this as a little grudging. Consequently, in what follows I treat my respondents’ talk not as clear examples of what they ‘truly’ believe are the implications of epigenetics, but instead as further occasions through which novelty is negotiated. When the scientists I spoke with did articulate the ethical and social dimensions of their work, they leveraged established scientific and bioethical tropes. I will illustrate this through sizable extracts from two interviews where the ethical implications of epigenetics emerged, before then indicating how these ramifications related to pre-existing discourses. First, R7, who after initially noting that their research did not raise any particular ethical issues then went on to say:

[O]ne of the goals [. . .] is to be able to figure out people’s biological age from their epigenome. And I guess that does have big *ethical* implications, because [. . .] if it were possible to do that through a blood test for example, then you’ve got insurance companies wanting to [. . .] figure out people’s biological age and their predisposition to disease from their epigenome, which obviously does have big ethical implications. [. . .] I don’t tend to think about it. None of the information we’re generating – you know, to me it will all be anonymised. None of it would be fed back to the [research participants]. In terms of the broader implications of figuring out this stuff [. . .] I believe that [. . .] knowledge should always be good provided it’s used in the right way. I guess we do have to decide how it’s gonna be used. I suppose I should probably be part of that debate.

R2 also spoke about the issues of insurance and risk prediction:

[I]f you can take a blood sample and that tells you about your risk of cancer, the same way [. . .] BRCA [gene] testing has just now, that’s self-evident. But there’s another spin to it I think with epigenetics that goes beyond that, which is [. . .] I talked about the example earlier of [how] we can distinguish by looking in at methylation and blood, between smokers and non-smokers but we can also distinguish between former smokers and current smokers or non-smokers, never smokers, and that again has implications [. . .] Obvious implications in terms of insurance but has other implications in terms of monitoring that kind of environmental exposure which I think [. . .] we’ve not particularly thought through yet.

Further:

I think the analogy with us mainly is [. . .] with the gene testing that’s been happening with say BRCA, as an example [. . .], and just now one can be in a situation where because of work that’s ongoing you discover that somebody is a BRCA carrier and I think [. . .] ethical advice around that has been very helpful in that *field*. And I think the same things could arise in the epigenetics area in a very analogous manner where [. . .] markers of risk or environmental exposure that we talked about get identified and then there is going to be an issue there about genetic counselling or epigenetic counselling! That’s going to have to be looked at where, advice from ethics would be absolutely essential.

In flagging risk prediction, R7 and R2 mirrored wider epigenetics discourse, wherein the notion of risk has traction. For instance, the authors of one review article in *Heredity* argued: ‘epigenetics holds substantial potential for furthering our understanding of the molecular mechanisms of environmental toxicants, as well as for predicting health-related risks due to conditions of environmental exposure and individual susceptibility’ (Bollati and Baccarelli, 2010: 105). The possibilities and perils of calculating *genetic* risk have circulated widely within the bioscientific, social scientific, bioethical and policy literatures for many years now (Hallowell, 1999; Novas and Rose, 2000). In this respect, the comments of R2 and R7 on the implications of epigenetics involve the reworking and interconnection of well-established scientific hopes and ethical concerns.

R4 also flagged a matter that resonates with longstanding concerns within and beyond the academy. Specifically, they spoke of ‘developmental programming’, whereby epigenetic processes in foetuses and neonates impact future health:

I think probably specifically the transgenerational thing [i.e. the possibility of inter-generational transmission of epigenetic marks] which is quite distinct and the, kind of, what you can also call developmental programming. So this means that things that have happened early in your life have an impact much later in your life. And whether it’s things that you’ve been given in nutrition during pregnancy, or scary things that happened in your early childhood. All this kind of stuff. So I think those are dimensions that are outside of genetics, at least in my perception I would say.

Despite being ‘outside of genetics’, such matters nevertheless resonate with wider scientific, policy, and health and social care practice-orientated concerns around the promotion of biopsychosocial flourishing in the early years of life, including the prenatal months (Gillies et al., 2016). Hence, while epigenetics research might indeed be ‘producing new versions of vulnerable, plastic life that require protection’ (Mansfield, 2017: 355), such vulnerabilities – and related ethical dilemmas about what to do about them – are well rehearsed. In a biomedical and cultural context in which statements like ‘things that have happened early in your life have an impact much later in your life’ form a common trope, the reflections of R4 are recognisable if not necessarily derivative. My point is that the issues raised by R4 do not appear to be indexical to this scientist’s specific research; instead, they are somewhat more general concerns raised in other contexts. Again, this is suggestive of the extent to which the implications of epigenetics are choreographed through rearranging components of pre-existing epistemic and normative projects.

To summarise, the ethical novelty of epigenetics was not taken for granted by the scientists I interviewed; rather, it was largely assembled through the sociological interview through prompts and following initial reflection. This novelty work entailed making links to debates and dialogues associated with other areas of bioscience, especially genetics. By making links to something old (namely, pre-existing science and/or ethical tropes), something new (i.e. the ethical implications of epigenetic research) could be built. Scientists’ talk about the wider ramifications of their work consequently provides a further window into how novelty is (de)constructed and negotiated.

## Conclusion

Ideas about epigenetics are beginning to inform social practices in a range of societies (Niewöhner et al., 2011; Warin et al., 2020). In relation to reproduction, for instance, epigenetic discourses have contributed to processes of responsibilisation (Pentecost, 2018; Richardson, 2015; Valdez, 2018). The various societal ramifications of epigenetics relate, in part, to the purported novelty of epigenetic knowledge. Rather than contributing directly to the literature on the social impacts of epigenetics, this article has examined *how* the novelty of epigenetics can be socially produced and negotiated. I suggest that this attention to novelty per se could have wider relevance to sociological scholarship.

To recap my findings and argument: first, I noted the definitional pluralism of ‘epigenetics’, arguing that this serves to produce and proliferate ascriptions of novelty (although I am mindful not to reduce definitional pluralism in general to being only or necessarily related to novelty). In the case of epigenetics, problematising the definition of the field helps to (re)present it as an important, new area of research that *requires* close characterisation. I suggest that any requirement is an achievement of definitional debate, rather than necessarily preceding it (Law, 1999; Mol, 1999). Subsequently, I examined how the novelty of epigenetics is synthesised in scientific texts and talk through its juxtaposition with genetics. The latter is often presented in such discourse as having epistemological limits that epigenetics might address. Epigenetics is thereby configured as new and vital, partly through the innovation it could enable. I then showed that the notion of epigenetics as having innovative potential was apparent within my interviews and in the biomedical literature. I linked notions of epigenetic innovation to excitement and intrigue, suggesting that the idea that epigenetics was new or might facilitate new developments was – in the emotional cultures of science – an accelerant of excitement. In turn, a position that epigenetics was exciting legitimates constructions of epigenetics as novel. Finally, I interrogated if and how epigenetics was configured as raising novel ethical issues. I showed how these were not taken for granted by scientists, and hence that the new can be deconstructed as well as assembled. Upon interview probes, my respondents occasionally articulated some ethical implications of epigenetics. Accounts often involved discursive links to pre-exiting debates or concerns (e.g. around risk and insurance) that provide an example of novelty work in the real-time of conversation: namely, the enrolment of something old to create something new. In sum, novelty is a discursive achievement that can be undone as well as assembled; it is negotiable, not quintessence.

An appreciation of the extent to which novelty is a social accomplishment has, I think, some implications for future sociological engagements with epigenetics. Within bioethics, Huang and King (2018: 77) have raised ‘caution against the temptation to claim that new research findings or technologies might unilaterally necessitate changes in ethical theory or policy recommendations’. Likewise, science studies scholar Sara Green (2016: 84) has highlighted the risk of a ‘non-critical endorsement’ of ‘breaking news about biological complexity’. Similarly, I tentatively suggest the need for hesitation in relation to any sociological embrace of epigenetics as a catalyst for redirecting social science and theory. Within sociological discourse, suggestions are beginning to be made that sociologists should intertwine epigenetic knowledge into conceptualisations about the ontologies of social practices. These are often undergirded by assertions that epigenetics is new, exciting and/or important. My point it not to dismiss knowledge-claims emanating from epigenetics, or to dissuade colleagues from biosocial theorisation. It is, rather, to emphasise that social scientific characterisations of a field as novel and important might be part of the machinery by which it becomes more widely regarded as such. Given aforementioned concerns about the implications of epigenetics for processes of, for instance, responsibilisation, there are reasons to be cautious about sociological contributions to novelty work in biomedicine and beyond.

The sociological analysis of novelty has the potential to make a wider contribution than solely to examinations of technoscientific innovation. Sociologists could inflect their work in ‘new’ ways through attention to how the actors and institutions they study negotiate different kinds of novelty. For instance, established debates about the construction of social problems and claims-making, and more recent conversations around post-humanism and new materialisms, might afford some benefits through a direct focus on the novel. Discourses, practices and entities are positioned as novel in a range of spheres, and attention to the material and semiotic work that goes into this can provide a further window into how action is enjoined or inertia maintained, generating a variety of insights into the questions of order, power and meaning that energise sociological praxis.

## Notes

1. As a consequence of the visibility of my respondents within epigenetics, and following discussions about confidentiality in the interviews, this article provides a level of anonymity which necessitates occlusions that could be dissatisfying to some readers. I give, for instance, few details about specific fields of research. These particularities, however, of course informed how I approached and treated the data – even though they might unfortunately be absent from the article itself.

2. In all quotes the italicised emphasis is in the original talk.

3. In my answer I acknowledged the diversity of definitions and noted that part of the interview would seek to elucidate what he himself regarded as ‘epigenetics’.

4. Notably, R5 slipped between descriptive and normative ascriptions of excitement in others: his comments above suggest that he both thought and hoped epigenetics would appeal to ‘the general public’.
